# Chromosome Location Contributing to Ozone Tolerance in Wheat

**DOI:** 10.3390/plants8080261

**Published:** 2019-08-01

**Authors:** Alsayed M. Mashaheet, Kent O. Burkey, David S. Marshall

**Affiliations:** 1 Department of Plant Pathology, Damanhour University, Damanhour 59, Egypt; 2 Department of Entomology and Plant Pathology, North Carolina State University, Raleigh, NC 27695, USA; 3 USDA-ARS, Plant Science Research Unit, 3127 Ligon Street, Raleigh, NC 27607, USA; 4 USDA-ARS, Plant Science Research Unit, 3411 Gardner Hall, North Carolina State University, Raleigh, NC 27695, USA

**Keywords:** wheat, *Triticum aestivum*, ozone, wheat genome

## Abstract

Breeding wheat for higher grain yield can contribute to global food security and sustainable production on less land. Tropospheric ozone can injure wheat plants and subsequently reduce grain yield. Identification of ozone tolerance in the wheat genome can assist plant breeders in developing new sources of tolerant germplasm. Our objective was to use the ‘Chinese Spring’ monosomic lines to screen for ozone response and identify the chromosomic locations contributing to ozone tolerance based on foliar injury. Two methodologies, Continuous Stirred Tank Reactors and Outdoor Plant Environment Chambers, were used to expose wheat monosomic lines to varying concentrations and durations of ozone. Each wheat monosomic line in ‘Chinese Spring’ has a missing chromosome in each of the wheat subgenomes (A, B, and D). In both methodologies, we found significant and repeatable data to identify chromosome 7A as a major contributor to tolerance to ozone injury in ‘Chinese Spring’. In every experiment, the absence of chromosome 7A resulted in significant injury to wheat due to ozone. This was not the case when any other chromosome was missing.

## 1. Introduction

Tropospheric ozone (O_3_) is a secondary air pollutant and a global warming gas in ambient air, the concentrations of which have been increasing since the industrial era and are expected to continue to increase in the future [1]. Once formed, the O_3_ molecules can be inhaled by humans and animals, causing respiratory problems, and can diffuse through stomata into plant leaves, where ozone is considered the most phytotoxic air pollutant [2]. Inside the leaf, O_3_ immediately dissolves, generating primary reactive oxygen species (ROS) in the apoplast [3]. If not detoxified, these non-biogenic ROS react directly in the apoplastic fluid with the cell wall and/or plasma membrane, inducing a self-propagating oxidative burst of biogenic ROS that spreads to the surrounding tissue [4]. The resulting oxidative stress causes visible injury and biomass and yield reduction in sensitive plants including major crops, such as wheat, soybean, corn, and rice, thereby threatening global food security.

Common bread wheat (*Triticum aestivum* L.) is one of the most sensitive crops to elevated O_3_ at all growth stages [5]. Modeling based on O_3_ concentrations in the atmosphere estimate current wheat yield reduction due to elevated O_3_ to be 7–12% of the yield worldwide, and this number is expected to reach 9–18% by the year 2030 [6,7]. Recent modeling work based on the more biologically relevant flux-based approach predicts similar yield reductions, but proposes a different geographical distribution of the impacts on wheat production as the result of regional differences in the environmental conditions that control O_3_ uptake through stomata [8,9]. Breeding wheat for O_3_ tolerance is essential for increasing yield per area under O_3_ stress, and is a critical adaptation needed to sustainably produce 70% more food by 2050 [10]. This is especially true in developing countries, where more food production is needed, and where O_3_ precursors emission and population are expected to continue increasing. For example, both the highest stomatal O_3_ fluxes and O_3_ concentrations are observed in the most populated region of the world, in South and East Asia [8,9]. In particular, resource-poor farmers in developing countries rely for food on new abiotic and biotic stress-tolerant varieties that are freely available.

Currently, O_3_ stress is not prioritized by wheat breeders [9,11,12]. This lack of breeding effort may be attributed to several factors. Wheat breeders are less familiar with O_3_ as a stress factor and the magnitude of O_3_ impacts on wheat. Research is lacking on the genetic control of O_3_ tolerance in wheat, including information on genetic markers associated with the trait and the best approaches for phenotyping key varieties and breeding lines. Breeding for O_3_ tolerance is further complicated by the temporal and spatial inconsistency of O_3_ stress, so that testing requires specialized research facilities.

The development of O_3_-tolerant wheat varieties can be accelerated if better information was available on the genetic control of the trait, and molecular markers are identified for easier and quicker identification of tolerant germplasm. The complex, allohexaploid (2*n* = 6*x* = 42 chromosomes, AABBDD) genome of common wheat became better understood with the availability of aneuploids, in particular monosomics, which allowed genes to be assigned to chromosomes [13]. In this work, we investigated the O_3_ response of ‘Chinese Spring’ and its monosomic series in order to identify the chromosome location of O_3_ response using visible foliar symptoms.

## 2. Results

### 2.1. Ozone Injury to ‘Chinese Spring’ Monosomics Using Continuous Stirred Tank Reactors

**Figure 1:**
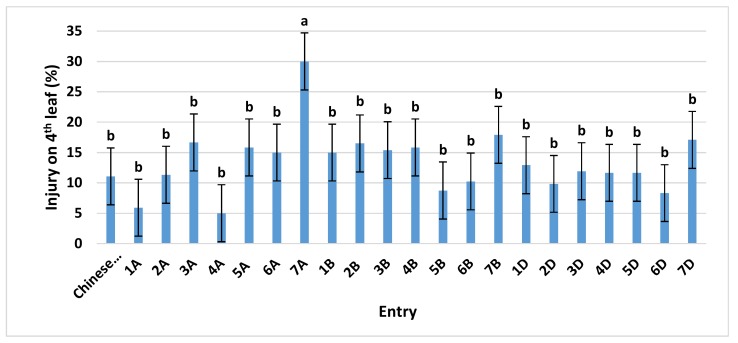
Ozone injury on the fourth leaf of ‘Chinese Spring’ and its 21 monosomic lines at four different O_3_ levels, exposed in Continuous Stirred Tank Reactors (CSTRs) for 5 days. Values are average ± standard error of 12 data points (four concentrations (50, 70, 90, and 110 ppb) × three blocks × one plant each) and are separated from the control (‘Chinese Spring’) using Dunnett’s test. For further details see Supplementary Figure S1.

Previous work [14] showed that ‘Chinese Spring’ would be a suitable indicator for O_3_ response because this variety exhibits a reduced O_3_-damage phenotype compared to other potential standard varieties. These results, coupled with the wealth of genotypic and phenotypic data on ‘Chinese Spring’, along with it being the reference variety for the sequencing of the wheat genome [15], make it ideal for O_3_ research. For our Continuous Stirred Tank Reactor (CSTR) study, ‘Chinese Spring’ and its 21 monosomic lines were first grown in charcoal-filtered (CF) air from planting until early tillering, Zadoks growth stage 21–23 [16]. Our CSTRs were located within an environmentally controlled greenhouse. At that time, the plants were moved to the five O_3_ treatments (CF, 50, 70, 90, and 110 ppb) for five days, and then two days after treatment they were visually assessed for O_3_ damage. Visual assessments were based on the relative percent of a leaf exhibiting chlorosis and necrosis. At the time of our assessments, the fourth leaf provided the most uniform growth stage. Averaged over O_3_ concentrations, ‘Chinese Spring’ averaged a mean O_3_ injury score of 11% (Figure 1).

The only monosomic line significantly different from ’Chinese Spring’ was the line missing chromosome 7A, at an O_3_ injury level of about 30%. When the responses of the monosomic lines were investigated within each O_3_ treatment, the monosomic line missing chromosome 7A always showed the greatest injury. However, the effect was significantly different from ‘Chinese Spring’ at only 110 ppb (Supplementary Figure S1). Thus, chromosome 7A was associated with increased tolerance to O_3_. Because other monosomic lines differed (though not significantly at the 95% confidence level) from ‘Chinese Spring’, it could be inferred that multiple minor genes/alleles may be associated with the O_3_ response in wheat, in addition to those major-effect gene(s) on chromosome 7A.

### 2.2. Ozone Injury to ‘Chinese Spring’ Monosomics Using Outdoor Plant Environment Chambers (OPECs)

**Figure 2:**
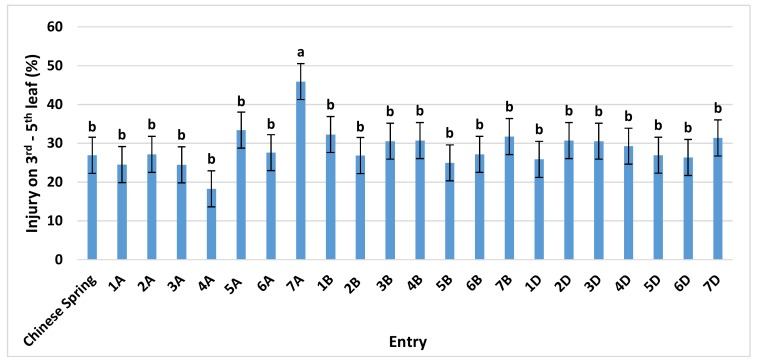
Ozone injury on the third to fifth leaf of ‘Chinese Spring’ and its 21 monosomic lines, at three different O_3_ levels, exposed for 14 days in the Outdoor Plant Environment Chamber (OPEC) system. Values are average ± standard error of 12 data points (three concentrations (50, 70, and 90 ppb) × two blocks × two plants each) and are separated from the control (‘Chinese Spring’) using Dunnett’s test. For further details see Supplementary Figure S2.

After growing to Zadoks growth stage 21–23, identical to the CSTR experiment, the plants were placed in OPECs at one of four O_3_ treatments (CF, 50, 70, and 90 ppb) for 14 days. Here, ‘Chinese Spring’ averaged an O_3_ injury of 27%, while monosomic 7A was again significantly higher at 46% (Figure 2). No significant differences were found with any of the B or D genome monosomics, or any other A genome monosomic line. Similar to the CSTR experiment, the absence of chromosome 7A always resulted in the greatest increase in O_3_ sensitivity under each of the three elevated O_3_ concentrations tested. However, the difference between 7A and ‘Chinese Spring’ was not significant under individual O_3_ treatments (Supplementary Figure S2).

## 3. Discussion

The goal of this study was to determine the individual chromosome contribution towards the known O_3_ tolerance in ‘Chinese Spring’ wheat. This objective was achieved by screening the cultivar with complete genome and its 21 monosomic lines under different O_3_ treatments, in two different gas exposure systems. Ozone injury data showed that chromosome 7A is critical for O_3_ tolerance in ‘Chinese Spring’. Our finding supports a previous report [17] attributing O_3_ tolerance in hexaploid wheat to subgenomes AABB. Biswas et al. [17] found that durum wheat (*Triticum durum*; AABB) was the most O_3_-tolerant, followed by *T. monococcum*, a successive sister of *T. urartu*, the more likely origin of genome AA. In this study, we provided direct evidence, using common bread wheat, that chromosome 7A has a major effect on the tolerance to O_3_ in ‘Chinese Spring’. The O_3_-induced injury reported here had a direct effect on reducing plant biomass [14], but further research is needed to relate the biomass reduction to grain yield.

Davydov [18] showed that the absence of chromosome 7A was associated with the least stomatal length amongst all monosomic lines, which indicates that the increased sensitivity observed in the absence of 7A reported in the present study is likely derived by other mechanisms that are not affected by the size of stomata. These mechanisms could include lower O_3_ detoxification or injury containment abilities. Interestingly, wheat chromosome 7A also carries other important stress- and disease-related traits, such as resistance to powdery mildew [19], salinity tolerance [20], and resistance to Fusarium Head Blight [21]. This research serves as an initial step in our identification of O_3_ tolerance in wheat. We are currently conducting a genome-wide association analysis of O_3_ injury data on a double-haploid population made between O_3_-sensitive and O_3_-tolerant wheat germplasms, with the goal of better pinpointing those genetic loci associated with O_3_ tolerance.

Evidence suggests that several mechanisms contribute to O_3_ tolerance in crops. For both rice [12] and soybean [22], multiple quantitative trait loci have been associated with O_3_ tolerance. It is reasonable to expect the same for wheat. Mills et al. [9] described a wheat ideotype with specific characteristics relevant to O_3_ stress responses. Characteristics include leaf traits that limit O_3_ uptake, metabolic traits that regulate antioxidant metabolism and programmed cell death, and root traits that enhance water and nutrient uptake under stress [9]. Each characteristic is potentially associated with different sets of markers. It is clear that yield superiority under O_3_ stress is the ultimate predictor for O_3_ tolerance. However, for screening large numbers of entries, biomass and visible symptoms are key indicators. Visible symptoms determine the active photosynthetic area, as well as the healthy host tissue interacting with O_3_ at later growth stages.

## 4. Materials and Methods

The ‘Chinese Spring’ monosomics were obtained from the USDA-ARS National Small Grains Collection in Aberdeen, ID. For both exposure systems, dry seeds were planted in 170 ml plastic containers (20.7 cm length, 4 and 2.5 cm diameter at the top and the bottom, respectively) filled with Fafard #2 Pro Mix (Fafard, Anderson, SC, USA). Plants were irrigated from the bottom in a water basin. Water soluble fertilizer (20–10–20 NPK) was applied to the water basin once a week. Seedlings were grown in CF air (O_3_ concentration = 5 ppb) in the greenhouse for 30 days after planting. Plants at an early tillering growth stage of 21–23 in Zadok’s scale were selected for growth stage and canopy status. The fully expanded fourth leaf on the top of the main stem was tagged and then plants moved to the Continuous Stirred Tank Reactors (CSTRs) [23,24] or the Outdoor Plant Environment Chambers (OPECs) [25].

In both systems, plants were acclimated for two days in CF before treatment with O_3_. In CSTRs, the five target O_3_ treatments (CF, 50, 70, 90, and 110 ppb) were planned for five days (7 h/day, at 25 °C and 60% relative humidity (RH)). The actual achieved treatments were 5, 49, 70, 92, and 107 ppb, respectively. The experiment design involved 15 chambers in three blocks. Each treatment was randomly assigned to one chamber per block. One plant per genotype per chamber was used. In OPECs, plants were exposed for 14 days to one of four O_3_ treatments using a predefined diurnal O_3_ profile (CF, 50, 70, 90 ppb, 12 h average, at 25/16 °C day/night and 50% RH). The actual achieved treatments were 5, 46, 65, and 84 ppb, respectively. Eight OPEC chambers divided into two blocks were used. Each treatment was randomly assigned to one chamber per block. Two plants per genotype were randomized to each chamber.

**Figure 3:**
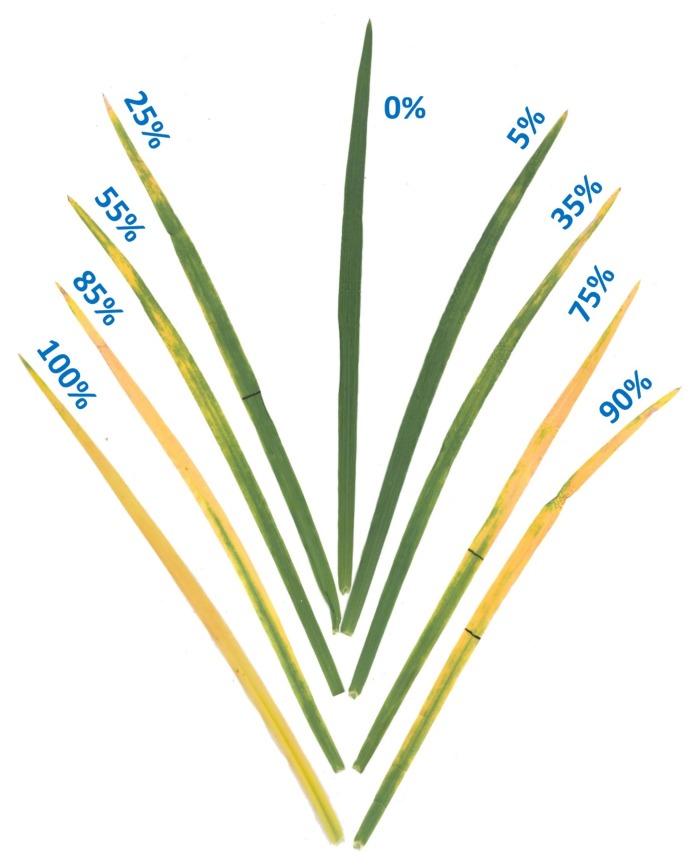
Ozone injury scale (0–100%) showing examples of different levels of O_3_ injury on leaves of winter wheat cultivar Coker-9553. Percent leaf injury levels were assessed using APS Assess 2.2 image analysis software after O_3_ exposure in the OPECs.

Foliar injury was assessed visually following a 0–100% scale (Figure 3) established using a color image analysis of scans of leaves showing different levels of O_3_ injury (Assess 2.2, The American Phytopathological Society, St. Paul, MN, USA). To assess O_3_ injury in the CSTRs, plants were assessed under constant light conditions two days after the end of exposure by percent O_3_ injury on the fourth leaf of the main stem, whereas, all leaves on the main stem of plants treated in OPECs were evaluated under constant light conditions after the last day of treatment. However, only the third to fifth leaves were considered for statistical analysis, as the first and second leaves showed some signs of senescence, and not all plants had fully expanded leaves above the fifth at the time of assessment. Data were subjected to analysis of variance using Glimmix procedure in SAS 9.4 (SAS Inc., Cary, NC, USA) as a split-plot design (O_3_ treatments in the main plot, and genotype in the subplot). Random effects of block and sampling were accounted for. Significant effects were determined at a significance level of 5% (*p* ≤ 0.05) and Dunnett’s test was used to compare each monosomic line to ‘Chinese Spring’.

## Supplementary Materials

The following are available online at https://www.mdpi.com/2223-7747/8/8/261/s1, Figure S1: Visible injury responses of spring wheat variety “Chinese Spring” and it’s monosomic lines exposed to different ozone levels in CSTRs. Figure S2: Visible injury responses of spring wheat variety “Chinese Spring” and it’s monosomic lines exposed to different ozone levels in OPECs.
