# Supplementary figures and images for: Chromosome Location Contributing to Ozone Tolerance in Wheat
Source: Plants (Basel). 2019 Aug 1;8(8):261. doi: 10.3390/plants8080261 (PMC6724167; doi:10.3390/plants8080261)

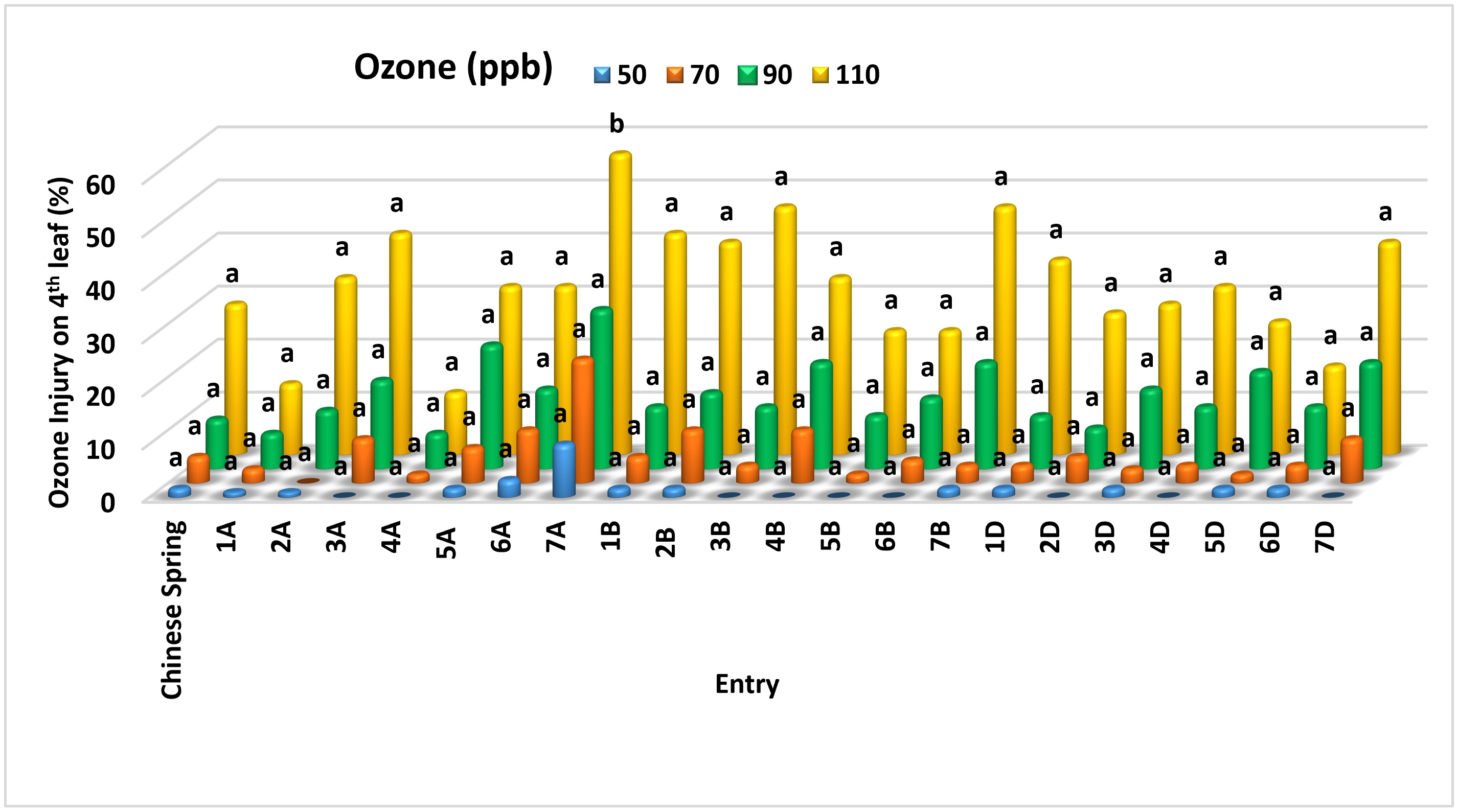

Supplement: Supplementary file 1 [file plants-08-00261-s001.zip › plants-534128-supplementary - r2/Supplementary Figure 1.jpg]

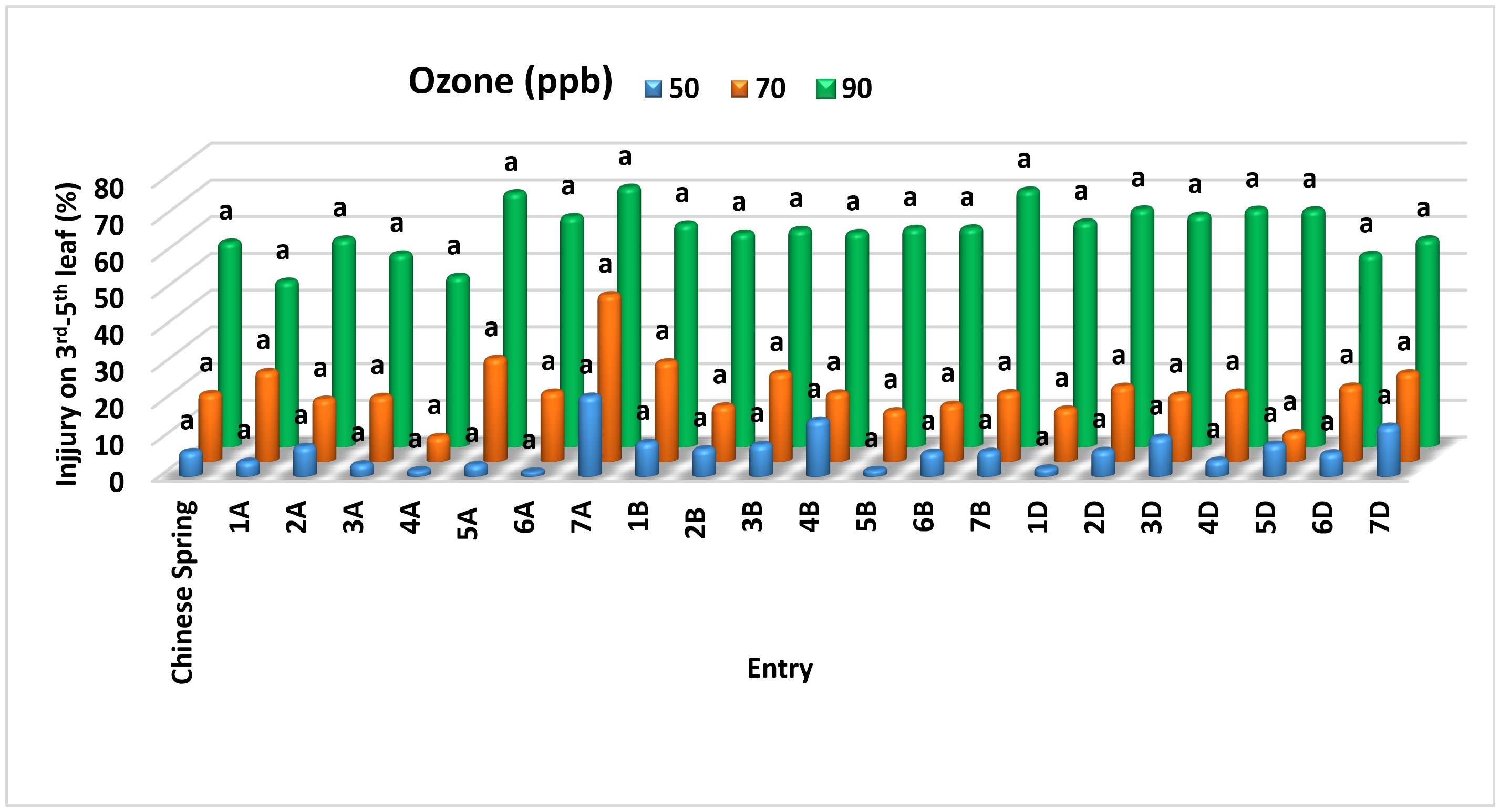

Supplement: Supplementary file 1 [file plants-08-00261-s001.zip › plants-534128-supplementary - r2/Supplementary Figure 2.jpg]
